# Antifungal and Antibacterial Activities of Isolated Marine Compounds

**DOI:** 10.3390/toxins15020093

**Published:** 2023-01-18

**Authors:** Amin Mahmood Thawabteh, Zain Swaileh, Marwa Ammar, Weam Jaghama, Mai Yousef, Rafik Karaman, Sabino A. Bufo, Laura Scrano

**Affiliations:** 1 General Safety Section, General Services Department, Birzeit University, Ramallah 00972, Palestine; 2 Faculty of Pharmacy, Nursing and Health Professions, Birzeit University, Ramallah 00972, Palestine; 3 Pharmaceutical Sciences Department, Faculty of Pharmacy, Al-Quds University, Jerusalem 20002, Palestine; 4 Department of Sciences, University of Basilicata, Via dell’Ateneo Lucano 10, 85100 Potenza, Italy; 5 Department of Geography, Environmental Management and Energy Studies, University of Johannesburg, Auckland Park Kingsway Campus, Johannesburg 2092, South Africa; 6 Department of European and Mediterranean Cultures, University of Basilicata, Via Lanera 20, 75100 Matera, Italy

**Keywords:** marine compounds, antibacterial activity, antifungal activity, minimum inhibitory concentration (MIC), bacterial resistance

## Abstract

To combat the ineffectiveness of currently available pharmaceutical medications, caused by the emergence of increasingly resistant bacterial and fungal strains, novel antibacterial and antifungal medications are urgently needed. Novel natural compounds with antimicrobial activities can be obtained by exploring underexplored habitats such as the world’s oceans. The oceans represent the largest ecosystem on earth, with a high diversity of organisms. Oceans have received some attention in the past few years, and promising compounds with antimicrobial activities were isolated from marine organisms such as bacteria, fungi, algae, sea cucumbers, sea sponges, etc. This review covers 56 antifungal and 40 antibacterial compounds from marine organisms. These compounds are categorized according to their chemical structure groups, including polyketides, alkaloids, ribosomal peptides, and terpenes, and their organismal origin. The review provides the minimum inhibitory concentration MIC values and the bacterial/fungal strains against which these chemical compounds show activity. This study shows strong potential for witnessing the development of new novel antimicrobial drugs from these natural compounds isolated and evaluated for their antimicrobial activities.

## 1. Introduction

Antibiotics are one of the most powerful medications developed to fight against dangerous infections. Their discovery has greatly improved human and animal health. Unfortunately, we are now witnessing a period in which people are dying from untreatable infections. The particular reason for these circumstances is the emergence and spread of antibiotic-resistant microorganisms. Antibiotic-resistant infections can be difficult, and sometimes impossible, to treat, resulting in mortality cases. The center for disease control and prevention CDC’s 2019 *Antibiotic Resistance (AR) Threats Report* mentions that antimicrobial resistance is an urgent global public health threat, killing at least 1.27 million people worldwide [1]. The report adds that more than 2.8 million antimicrobial-resistant infections occur each year in the U.S.A., causing the death of more than 35,000 people.

Antibacterial resistance is rapidly developing in bacteria as a result of the incorrect and excessive use of antibacterial medications among healthcare professionals and patients [2,3,4]. Therefore, there is a need to improve the appropriate use of antibiotics and reduce unnecessary use. Parallel to that, discovering and developing new antimicrobial products is of great importance to human, animal, and agricultural health [5].

The majority of new antibacterial agents developed in the pharmaceutical industry have been semisynthetic modifications of the original natural product discovered more than 50 years ago. In fact, beta-lactams, macrolides, and quinolones accounted for more than 70% of antibacterial medications approved between 1981 and 2005 [2]. Existing natural products that have been modified have given rise to compounds that momentarily defeat the resistance mechanisms. Eventually, bacterial resistance will be overcome only through the development of completely new natural compounds.

Over the past 50 years, most antibiotics have been discovered in terrestrial species. The ability of aquatic animals to create antibacterial chemicals has received little attention. The marine ecosystem is the largest and most important ecosystem on earth. It includes a great diversity of different groups of organisms that range in size from nanoscale microorganisms to whales [6]. Therefore, the ocean represents a potential source for the development of new antibiotics, and research into uncharted marine ecosystems is necessary to meet the pressing demand for new effective antibiotics. This variety provides a wealth of sources for the exact purpose of identifying novel medications that may be effective against particular diseases. There is a substantially higher chance of finding new antibacterial drug leads in marine environments than in terrestrial environments [7].

A variety of marine organisms, including bacteria, fungi, seaweeds, corals, sea cucumbers, sponges, and others, have been used to isolate antifungal and antibacterial biological compounds that fall into the following chemical groups: peptides, terpenoids, diacylglycerols, steroids, polysaccharides, polyketides, alkaloids, and others [8,9,10,11,12,13].

Although utilizing the reservoir of marine species freely for bioassays and therapy is difficult due to the relatively low availability of biologically active compounds, these difficulties can be overcome by several methods, such as mariculture (the cultivation of marine sponges), sponge-bioreactor specimen creation, sponge-cell culture systems (perimorph culture), genetic modification, and synthesis. The preferred options among them are still chemical synthesis and semi-synthesis. To investigate structure-activity relationships (SAR), synthetic organic chemistry can offer extensive biological screening and access to synthetic analogs [14,15].

The present study aims to investigate and summarize the antifungal and antibacterial activities of different chemical compounds isolated from marine organisms.

## 2. Isolated Marine Compounds with Antifungal Activity

### 2.1. Antifungal Compounds Isolated from Marine Bacteria

Marine microbes, frequently referred to as chemical gold, are considered to be a great source of novel treatments [16,17]. Bacteria are ubiquitous throughout the marine ecosystem. They can adapt to and change for any challenging environment. Therefore, marine bacteria are generally more effective than terrestrial bacteria in the bioremediation of toxic, heavy metals, hydrocarbon, and xenobiotics, as well as many other recalcitrant compounds. This is attributed to the production of extracellular polymeric substances (EPS) and the formation of biofilms [18].

**Scheme 1:**
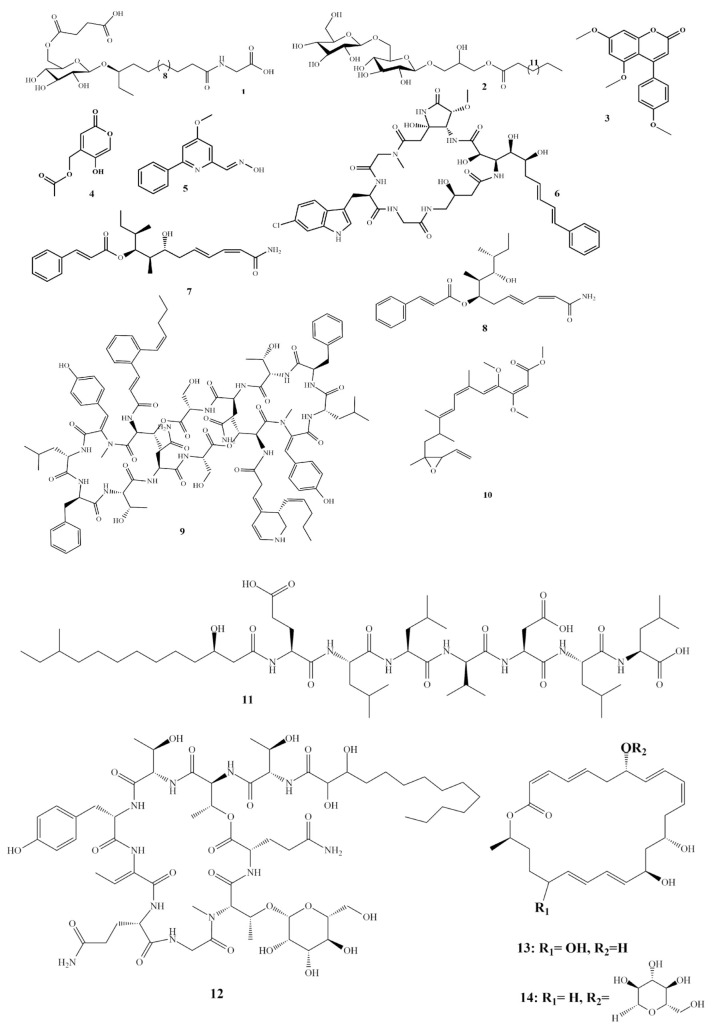

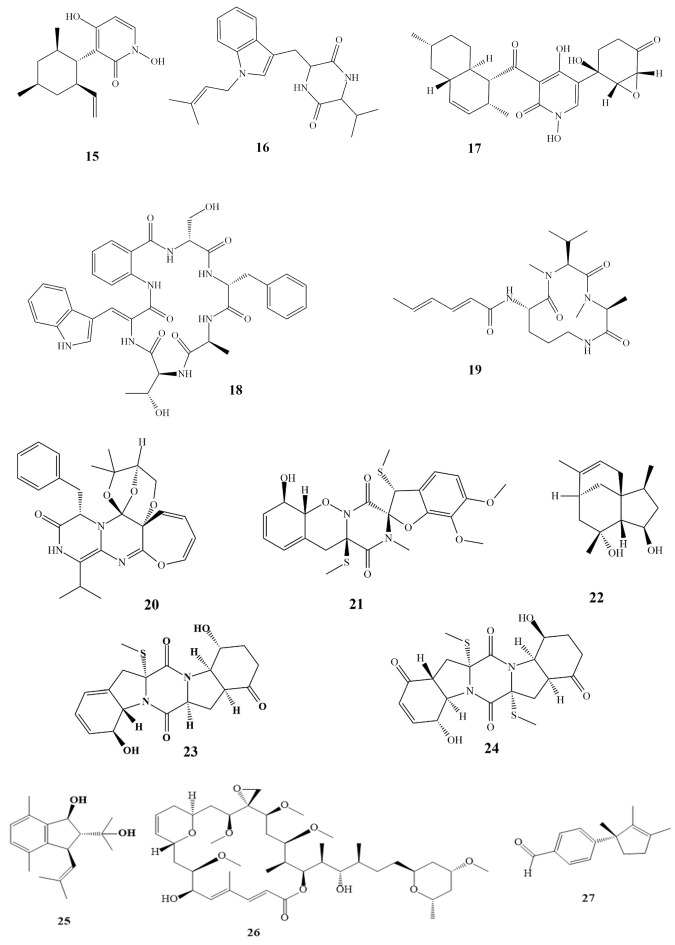

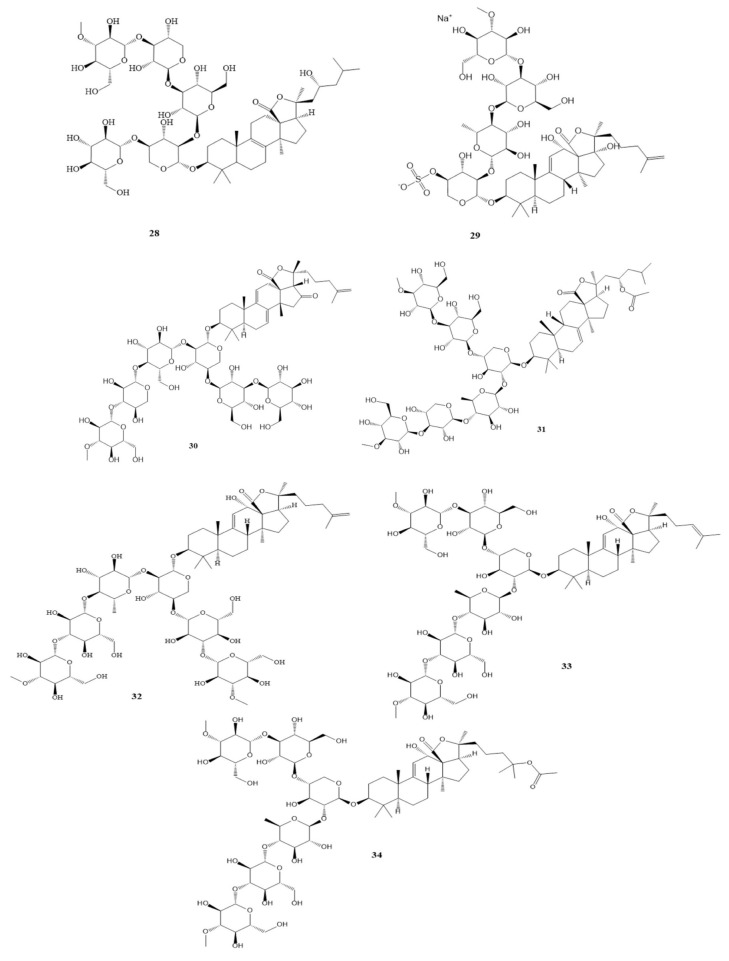

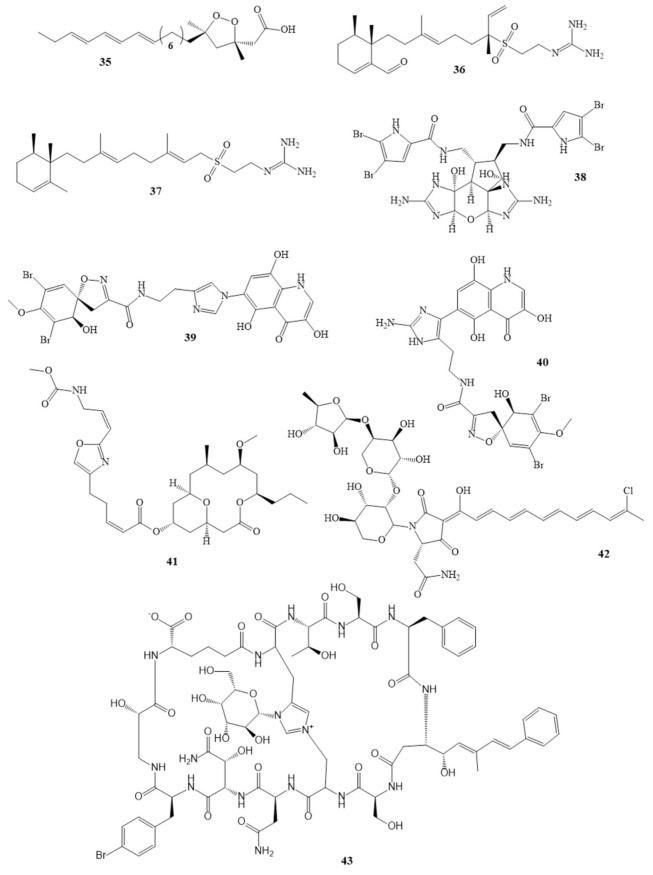

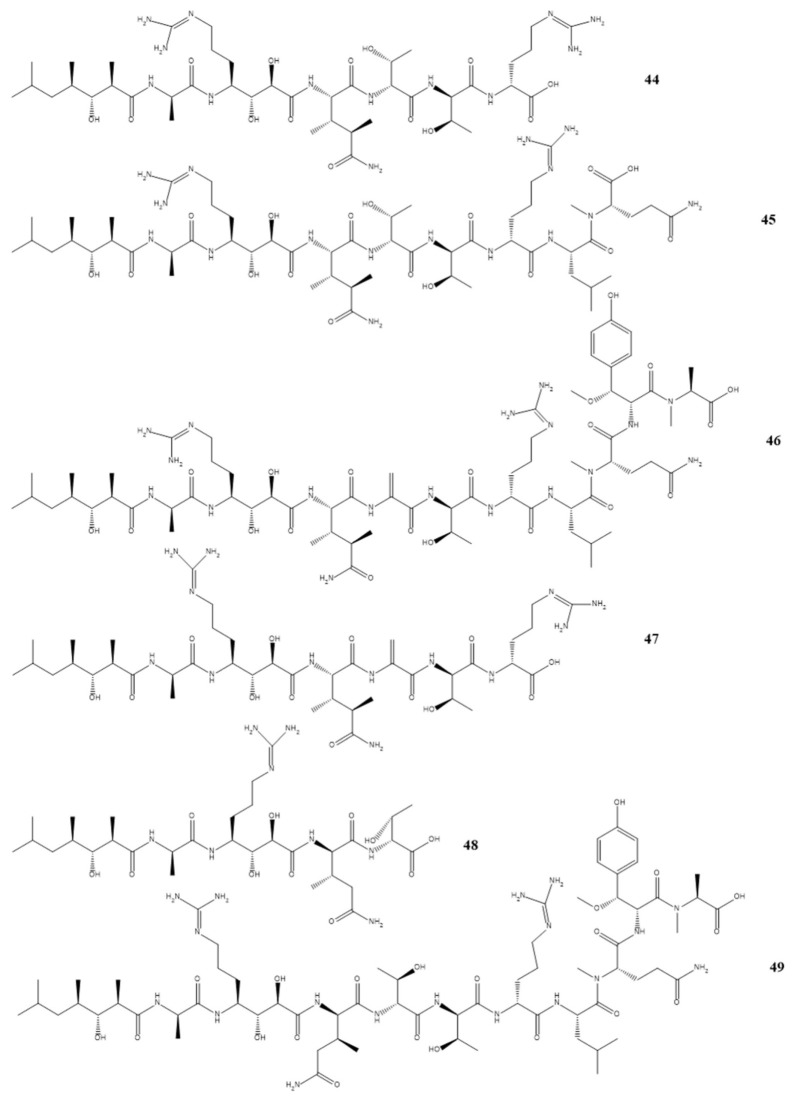

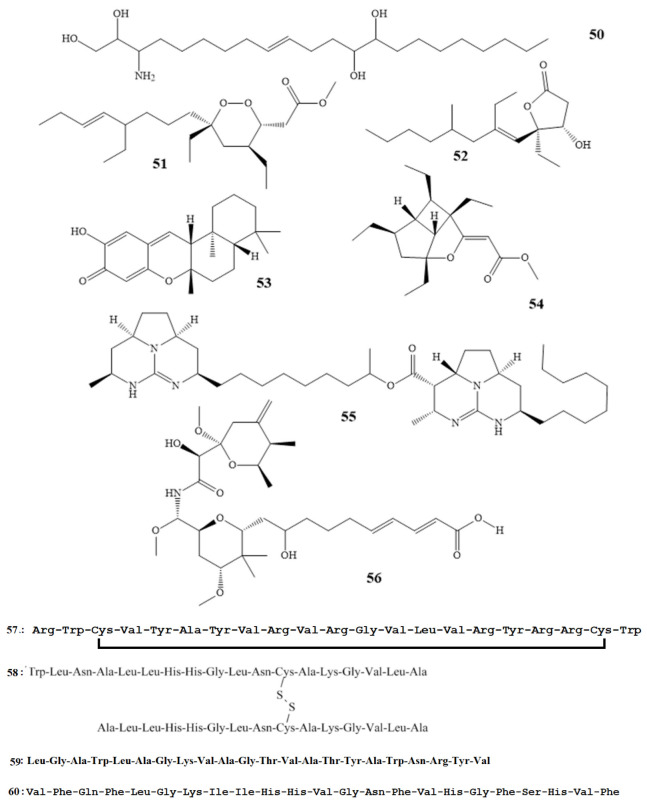

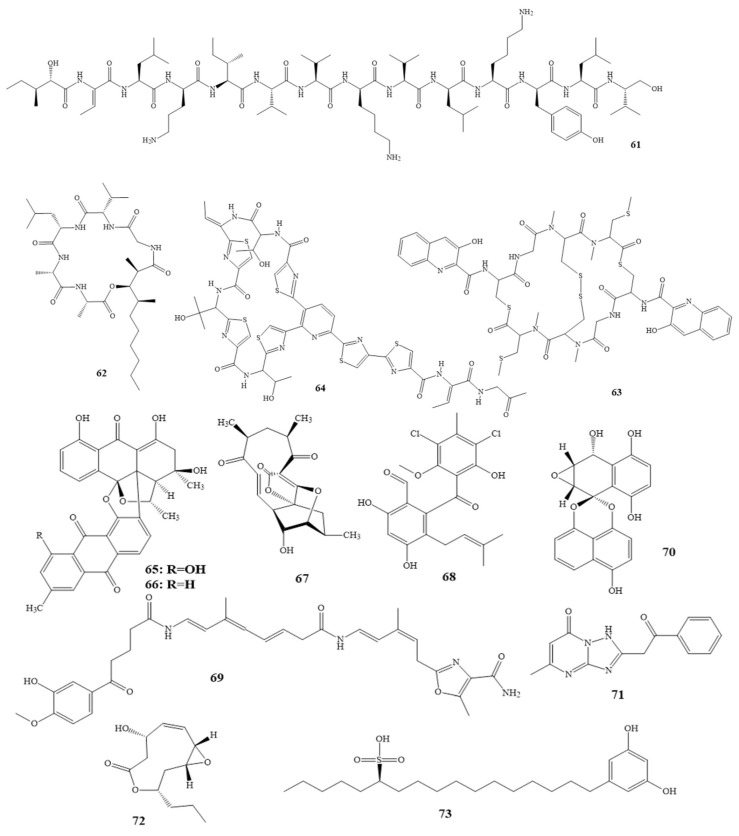

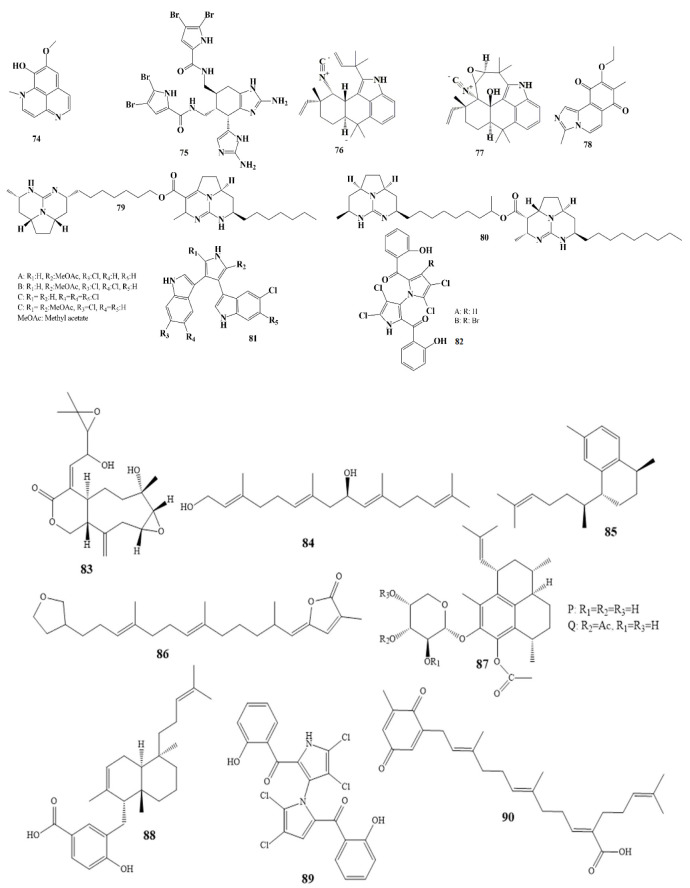
Chemical structures of cited compounds that were isolated from marine organisms and that showed antimicrobial activities.

Ieodoglucomide C (**1** in Scheme 1) and ieodoglycolipid (**2** in Scheme 1) are two glycolipids which are both isolated from the aquatic bacterium *Bacillus licheniformis*. It was found that they both have potent antifungal activity, with MIC values of 0.02–0.03 µM against the human pathogens *Candida albicans*, *Colletotrichum acutatum*, *Botrytis cinerea*, *Rhizoctonia solani*, and *Aspergillus niger* [19,20].

Hedaya48, which was synthesized by the *Aplysina fistularis* sponge when subjected to various UV radiation dosages, 5,7-dimethoxy-4-p-methoxyl phenyl coumarin (**3** in Scheme 1), and saadamycin (**4** in Scheme 1) were all new antimycotic substances identified from endophytic *Streptomyces* sp. The MIC value of saadamycin was reported to be 1–5.16 µg/mL, whereas 7.5–100 µg/mL was observed for 5,7-dimethoxy-4-p-methoxyl phenyl coumarin against dermatophytes as well as other fungi, including *Cryptococcus humicolus*, *Fusarium oxysporum*, *Aspergillus fumigatus*, *A. niger*, and *Microsporum gypseum* [21,22].

Actinomycetes were used to create the new and superior antifungal drug caerulomycin A. (**5** in Scheme 1). Actinomycete strain PM0525875 for extraction was obtained from a marine invertebrate. Actinomycetes extracts showed strong effectiveness against drug-resistant fungus strains in in vitro investigations. The fluconazole-resistant *Candida glabrata*, *C. albicans*, *C. albicans CO9*, and *Candida krusei* were the pathogenic fungal test strains used to determine the MIC value of caerulomycin A. The MIC values reported ranged between 0.39 and 1.56 µg/mL [23,24].

The secondary metabolite, pedein A (**6** in Scheme 1), was isolated from the cell mass of the myxobacterium *Chondromyces pediculatus*. Pedein A inhibited the growth of a broad spectrum of yeasts and fungi, whereas Gram-positive and Gram-negative bacteria such as *Bacillus subtilis*, *Brevibacterium ammoniagenes*, *Corynebacterium fascians*, *Micrococcus luteus*, *Staphylococcus aureus*, *Escherichia coli*, *Enterobacter aerogenes*, *Pseudomonas aeruginosa*, and *Salmonella typhimurium* were not sensitive to the antibiotic. MIC value for *Rhodotorula glutinis* was reported to be 0.6 µg/mL, and an MIC value of 1.6 µg/mL was reported for both *Saccharomyces cerevisiae* and *Candida albicans*. Furthermore, pedein A showed inhibitory activity against the growth of some filamentous fungi with a zone diameter range of 22–35 mm for *Botrytis cinerea*, *Gibberella fujikuroi*, *Pythium debaryanum*, *Rhizopus arrhizus*, *Trichoderma koningii*, and *Ustilago maydis* [20,21].

Other important isolated antifungal compounds, their marine sources, and their activities are listed in Table 1.

### 2.2. Antifungal Compounds Isolated from Marine Fungi

From the ocean’s surface to its deepest parts, fungi have been discovered to exist in almost every aquatic habitat studied [25]. As a result of marine fungi’s superior biological characteristics to terrestrial fungi and their ability to adapt to extreme pH, temperature, and salinity, a wider range of biotechnological applications of marine fungi are possible [26].

In Greenland, *Trichoderma* sp. strain MF106 was the source of pyridoxatin (**15** in Scheme 1), demonstrating antifungal activity with IC_50_ values of 1.07 ± 0.34 µM against *Trichophyton rubrum* and 6.9 ± 0.04 µM against *C. Albicans* [27,28]. The Japanese isolated diketopiperazine (**16** in Scheme 1) demonstrated growth inhibition against *P. oryzae.* and *P. yezoensis* with an IC_50_ value of 350 nM [29,30].

Didymellamide A (**17** in Scheme 1), isolated from the fungus *Stagonosporopsis cucurbitacearum*, reduced the growth of *C. albicans*, *Candida glabrata*, and *Cryptococcus neoformans* strains at doses of 1.6–3.1 µg/mL [31,32]. Additionally, the *Aspergillus sclerotiorum* PT06-1 isolates of sclerotide B (**18** in Scheme 1) and sclerotide B (**19** in Scheme 1) both exhibited activity against *Candida albicans*, with MIC values of 7.0 and 3.5 µM, respectively [33,34].

The plant pathogenic fungus *Fusarium graminearum*, *Alternaria brassicae*, and *Colletotrichum gloeosporioides* were all inhibited by varioxepine A, which has an MIC value of 4 µg/mL; peniciadametizine A, which has an MIC value of 4 µg/mL; and penicibilaenes A, which has an MIC value of 1.0 µg/mL (**20**–**22** in Scheme 1), respectively. They were extracted from *Paecilomyces variotii*, *Phoma* sp. Q60596, and *Penicillium bilaiae* MA-267 fungus [35,36,37]. On the other hand, penicibrocazines B and E (**23**,**24** in Scheme 1), which were isolated from *Penicillium brocae* MA-231 (*Avicennia marina* culture extract), showed activity against the plant pathogen *Gaeumannomyces graminis*, with a 0.25 µg/mL MIC value for both [38].

### 2.3. Antifungal Compounds Isolated from Marine Algae

Caulerprenylol B (**25** in Scheme 1), which was obtained from Chinese alga *Caulerpa racemosa*, has excellent antifungal activity against *T. rubrum* fungus, which causes two of the most common fungal infections, known as ’athlete’s foot’ and ’jock itch’, with an MIC_80_ value of 16 µg/mL [39].

Lobophorolide (**26** in Scheme 1), isolated from *Lobophora variegata* (marine brown alga) of the Bahamas and Egypt, has excellent activity against the pathogenic ascomycete *Lindra thalassiae* and the saprophytic deuteromycete *Dendryphiella salina*, with IC_50_ values of 0.135 and 0.034 µg/mL, respectively. Further, it showed antifungal activity against *C. albicans* wild and amphotericin-resistant strains, with IC_50_ values of 1.3 and 0.5 µg/mL [24,40].

The isolated isolauraldehyde (**27** in Scheme 1) showed antifungal activity against *C. albicans*, *A. fumigatus*, and *A. flavus* with MIC values of 70, 100, and 1000 µg/mL, respectively. The organic extract of isolauraldehyde was obtained from the red alga *Laurencia obtuse* [41,42].

The growth of *Mycobacterium smegmatis* and *Neurospora crassa* could be inhibited by the ethanolic extract of *Gracilaria domigensis* [43]. *Gracilaria sjoestedii* and *Gracilaria debilis* ethanolic extract had antifungal activity against *C. albicans* [44].

### 2.4. Antifungal Compounds Isolated from Sea Cucumbers

Sea cucumbers are animals with long bodies and leathery skin. They contain several antifungal compounds, such as variegatuside D. (**28** in Scheme 1), which was isolated from *Stichopus variegates* and which showed antifungal activity against *Microsporum gypseum*, *C. albicans*, *C. pseudotropicalis*, and *C. parapsilosis*, all of which have 3.4 µg/mL MIC_80_ value [45,46].

Scabraside A (**29** in Scheme 1) isolated from *Holothuria scabra* exhibited antifungal activities against *A. fumigatus, C. pseudotropicalis*, *M. gypseum*, *T. rubrum*, and *C. albicans*, with MIC values of 2, 4, 4, 8, and 8 µg/mL, respectively [47].

Antifungal activity against *C. tropicalis* and *M. gypseum* 31388 with MIC_80_ values of 1.4–5.7 µM were reported for holotoxin D1 (**30** in Scheme 1) and stichloroside C1 (**31**, in Scheme 1), which were isolated from *Apostichopus japonicus* Selenka [48].

The growth of *Cryptococcus neoformans*, *Richophyton rubrum*, *C. albicans*, *C. tropicalis*, *A. fumigatus*, and *C. krusei* could be inhibited with MIC_80_ values ranging from 0.7 to 2.81 µM by marmoratoside A, impatient side A, and bivittoside D (**32**–**34** in Scheme 1) isolated from *Bohadschia marmorata* Jaeger [49,50].

### 2.5. Antifungal Compounds Isolated from Sea Sponges

Sponges are elementary multi-cellular animals with dense skeleton muscles. They have a vast repertoire of antifungal compounds, which are useful in cases of resistance to amphotericin B and fluconazole [51].

The growth of *C. albicans* was inhibited by the isolated epiplakinic acid F (**35**, in Scheme 1) and agelasidine F and C (**36**,**37** in Scheme 1), which have MIC values of 3.1, 4, and 0.5 µg/mL, respectively. Epiplakinic acid F was extracted from the Seychelles sponge genus *Plakinastrella*. Agelasidine F and C were obtained from *Agelas citrina* (Caribbean sponge) [51,52,53]. Table 2 lists other isolated compounds from sea sponges that exhibit antifungal activity against *C. albicans*.

The highly oxygenated alkaloid massadine (**38** in Scheme 1), which was isolated from the marine sponge *Stylissa aff. massa*, inhibited Geranylgeranyltransferase-I from *C. albicans* with an IC50 value of 3.9 µM. Moreover, massadine inhibited the growth of *Cryptococcus neoformans* with an MIC value of 32 µM, but it did not inhibit the growth of *C. albicans* at a concentration of 64 µM [53].

Haliscosamine (**50** in Scheme 1), plakortide F acid (**51** in Scheme 1) and simplexolide E (**52** in Scheme 1) showed antifungal effectiveness against *C. neoformans* with MIC values ranged 0.2–3.66 μg/mL, respectively.

Haliscosamine was obtained from *Haliclona viscosa* (Moroccan sponge), plakortide F acid from *Plakortis halichondrioides* sponge, and simplexolide E from the sponge *Plakortis simplex* found in China [62,63,64].

Puupehenone (**53** in Scheme 1) (isolated from *Hyrtios* sp. sponge) showed antifungal activity with MIC values of 1.25 µg/mL and 2.50 µg/mL against *C. neoformans* and *C. krusei*, respectively [65]. The isolated Chinese Hippolachnin A (**54** in Scheme 1) from *Hippospongia lachne* sponge showed antifungal activity against *C. neoformans*, *T. rubrum*, and *M. gypseum*, with MIC values of 0.41 μM for each fungus [66]. Furthermore, with MIC values ranging between 1.9 and 7.8 µg/mL, the Brazilian batzelladine L (**55** in Scheme 1) isolated from the *Monanchora arbuscular* sponge exhibited activity against *A. flavus* strains [67].

A reasonably new nematicide (a substance active against nematode worms), onnamide F (**56** in Scheme 1), which was isolated from *Trachycladus laevispirulifer*, is helpful in *Saccharomyces cerevisiae* or baker’s yeast infections. It has an LD_99_ (dosage required to kill 99% of the fungi population) of 1.4 μg/mL [68].

Fluconazole resistance has been increasing recently, specifically in immunocompromised individuals such as HIV patients prescribed fluconazole prophylactically. Because of that, other antifungal compounds have been screened for efficacy in resistant strains. Geodisterol-3-O-sulfite and 29-demethylgeodisterol-3-O-sulfite, active constituents of *Topsentia* sp. extracts, have been used in fluconazole-resistant strains. Many *Saccharomyces cerevisiae* strains can overexpress the MDR1 efflux pump (a pump responsible for pumping out toxic substances such as fluconazole). Hence, these two compounds have been used in reverse [69].

## 3. Isolated Marine Compounds with Antibacterial Activity

### 3.1. Ribosomal Peptides—Antimicrobial Peptides

Antimicrobial peptides (AMPs) are large, amphipathic molecules synthesized by ribosomes using 12–45 amino acids, which typically have a tertiary structure (conformation). Due to their broad-spectrum antibacterial properties, they are suited for targeting prokaryotic cell membranes. AMPs are different from the adaptable lymphocyte-based immunity that characterizes higher vertebrates. AMPs that are produced by bacteria are named bacteriocins. In multicellular organisms, AMPs are found on the external surfaces (skin) or within the neutrophils. Marine invertebrates have their AMPs in cells that are similar to neutrophiles called hemocytes. Due to the presence of a good amount of lysine, arginine, and histidine and a low amount of acidic and neutral amino acids, AMPs are highly cationic at physiological pH. In addition to possessing phospholipids with no net charge, this cationic nature gives AMPs selectivity and selective toxicity towards bacterium cells, and their amphipathic nature may help explain their antibacterial effect [5,70,71].

Arenicin-1 is a peptide that is purified from the hemocytes of lugworm *Arenicola marina* [72]. The linear sequence polypeptide is composed of RWSIVYAYVRVRGVLVRYRRSIW, with a positive 6 net charge (**57** in Scheme 1) [73]. Arenicin-1 (**1**) inhibited Gram-negative bacteria such as *Escherichia coli* and *Proteus mirabilis* and Gram-positive bacteria such as *Staphylococcus aureus* with MIC values of 0.8, 2, and 6 µg/mL, respectively [74,75].

Halocidin is derived from the tunicate *Halocynthia aurantium*’s hemocytes [76]. The translated peptide consists of a halosidine segment, a single glycine residue, an N-terminal signal peptide, a C-terminal anion extension, and a single glycine residue (**58** in Scheme 1) [77]. On the other hand, the modified active peptide comprises two peptides, one with 15 amino acids and the other with 18, joined by a disulfide bond. Halocidin congeners, called Khal, appeared to have potent antibacterial action against methicillin-resistant *Staphylococcus aureus* (MRSA), vancomycin-resistant *Enterococcus* (VRE), and segregates of polyresistant *Pseudomonas aeruginosa* with MICs within the range of 2–4 μg/mL. One of two derivatives showed promising results in an animal model of *Listeria monocytogenes* infection [78].

Hedistin (**59** in Scheme 1) is an amphipathic antibacterial polypeptide obtained from *Nereis diversicolor* coelomocytes, a marine annelid worm [79]. This peptide had a high MIC of 1–2 μg/mL against *Micrococcus luteus* and *Micrococcus nishinomiyaensis*, indicating that it was effective against Gram-positive bacteria. The synthesized peptide was effective against *S. aureus*, with MIC values ranging from 8 to 15 μg/mL, as well as other *Staphylococcus* species [80].

Clavanin A (**60** in Scheme 1) peptide that isolated from the hemocytes of the *Styela clava*. [81,82] Clavanin A is rich in phenylalanine amino acids, which can replace it with other hydrophobicity amino acids without losing antibacterial activity [83]. Clavanin A showed potent antibacterial activity against Gram-positive as well as Gram-negative bacteria [84]. Clavanin’s MIC against *S. aureus*, including methicillin-resistant *S. aureus* strains, equals 1.4 to 3.8 μg/mL. Three strains of *Enterococcus faecium* had MIC values ranging from 0.1 to 1.1μg/mL. Strains of *E. coli* with an MIC value ranging from 0.4 to 2.3 μg/mL and three strains of *P. aeruginosa* with an MIC value ranging from 0.4 to 0.8 μg/mL were likewise susceptible to clavanin A [85,86].

### 3.2. Nonribosomal Peptides

Large multifunctional protein complexes called nonribosomal peptide synthetases (NRPSs) produce nonribosomal peptides [87]. The DNA does not encode many nonproteinogenic amino acids in these peptides. The most common nonribosomal peptides with antibacterial activity include bogorol A (**61** in Scheme 1), which was isolated from *Bacillus laterosporus* bacterium. It was confirmed that bogorol A showed activity in opposition to Methicillin-resistant *S. aureus* with an MIC of 2.5 μg/mL as well as vancomycin-resistant *Enterococcus* with an MIC of 9 μg/mL [88]. The cationicity of bogorol A has a significant role in targeting the bacterial membrane.

With an MIC value of 2.3 μg/mL, the isolated emericellamide A (**62** in Scheme 1) from the marine fungus *Emericella* sp. exhibited antibacterial activity against *S. aureus* [89]. Furthermore, thiocoraline (**63** in Scheme 1), which was isolated from *Actinomycete micromonospora*, showed activity against *S. aureus*, *M. luteus*, and *B. subtilis* with MIC values of 0.03–0.05 μg/mL [90,91].

Bleich et al. [92] described YM-266183 (**64** in Scheme 1) as an antibacterial peptide. It was produced by *Bacillus cereus* isolated from a marine sponge *Halichondria japonica*. The peptide is highly active against Gram-positive bacteria, including *S. aureus* and *Enterococci*, with MIC values of 0.68 μg/mL and 0.025 μg/mL, respectively [92,93].

### 3.3. Polyketides

Bisanthraquinone metabolites BE-43472B and BE-43472A (**65**,**66** in Scheme 1) isolated from a marine streptomycete showed biological activities against clinically derived isolates of *E. faecium* as well as *S. aureus*. The most potent activity displayed MIC values of 0.23 and 0.90 µg/mL against a panel (*n* = 25 each) of clinical MRSA and VRE, respectively [10,94].

The obtained Abyssomicin C AB 18-032 (**67** in Scheme 1) from marine actinomycete *Verrucosispora* sp. [10] exhibits potent antibiotic activity against Gram-positive bacteria, including pathogenic *S. aureus* strains, with an MIC value of 4 μg/mL [95].

Pestalone (**68** in Scheme 1), which is produced by a cultured marine fungus isolated from the brown alga *Rosenvingea* sp., showed potent antibiotic activity against methicillin-resistant *S. aureus* as well as vancomycin-resistant bacteria with MIC values of 37 µg/mL and 78 µg/mL, respectively [96,97]. Table 3 lists some other isolated polyketide compounds.

### 3.4. Alkaloids

An aaptamine—that is, a 1H-benzo[de][1,6]-naphthyridine alkaloid, Isoaaptamine (**74** in Scheme 1)—was isolated from the marine sponge *Aaptos aaptos* and was evaluated as a potent inhibitor with an IC_50_ value of 3.7 ± 0.2 µg/mL against *S. aureus* [105].

The bromopyrrole alkaloid nagelamides G (**75** in Scheme 1), which was isolated from the Okinawan marine sponge *Agelas* sp., exhibited antibacterial activity against Gram-positive bacteria *M. luteus* and *B. subtilis* with MIC values of 2.08 and 16.7 µg/mL, respectively [106].

With MIC values of 0.625 and 1.25 µg/mL, ambiguine H isonitrile (**76** in Scheme 1) obtained from *Fischerella* sp. showed activity against *Scaphirhynchus albus* and *B. subtilis*, respectively. Furthermore, ambiguine I isonitrile (**77** in Scheme 1) exhibited antibacterial inhibitory activities against the same bacterial strains with MIC values of 0.078 and 0.312 µg/mL, respectively [107]. Furthermore, cribrostain 6 (**78** in Scheme 1), which was isolated from the blue marine sponge *Cribrochalina* sp., showed an antibacterial activity against the same bacterial strain with MIC values of 16 and 2 µg/mL, respectively [108].

*Staphylococcus aureus* (methicillin-resistant *S. aureus*) and *Mycobacterium intracellulare* were both inhibited with MIC values of 5 and 10 µg/mL, respectively, by batzelladine M (**79** in Scheme 1). Additionally, antibacterial inhibitory activities against *P. aeruginosa* were also shown by batzelladine L (**80** in Scheme 1) with MIC values ranging from 0.31 to 20 µg/mL. Batzelladine L and batzelladine M are two polycyclic guanidine alkaloids extracted from the Jamaican sponge *Monanchora unguifera* [109]. Antibacterial inhibitory activities were also ascertained for lynamicins A-D (**81** in Scheme 1), which were isolated from a marine actinomycete, NPS12745, with MIC values ranging between 1.8 and 9.5 µg/mL [110].

Marinopyrroles A and B (**82** in Scheme 1), which were both isolated from marine *Streptomyces* strain bacterium, exhibited strong antibiotic activities against MRSA, with an MIC value range of 0.31–0.61 µg/mL [111].

### 3.5. Terpenes

Terpenes are a diverse class of natural products composed of repeating isoprene units. They include hemiterpenes (C5), di-unit monoterpenes (C10), tri-unit sesquiterpenes (C15), tetra-unit diterpenes (C20), penta-unit sesterterpenes (C25), and so on. They are created when mono-isoprene is broken down one unit at a time. Skeletal rearrangements, which frequently take place, alter the normal head-to-tail orientation of the isoprene units and add variety to the terpenoid structures [112].

The isolated xeniolide I diterpenes (**83** in Scheme 1) from soft coral *Xenia novaebrittanniae* shows activity against *B. subtilis* and *E. coli* with MIC values of 1.00 and 1.25 µg/mL, respectively [113,114].

The acyclic diterpene, crinitol (**84** in Scheme 1), which was obtained from *Sargassum tortile* alga, exhibits antibacterial activity against *Propionibacterium acnes*, *B. subtilis*, and *Streptococcus mutans* with MIC values of 25, 50, and 50 µg/mL, respectively [115].

More terpenes extracted from marine organisms are summarized in Table 4.

## 4. Conclusions

To sum up everything that has been stated so far, this review shed light on 96 compounds isolated from a variety of marine organisms and showed promising activities against bacteria and fungi. These compounds show great potential for the development of novel antibiotic drugs that can help overcome the problem of antibiotic resistance and have the potential to decrease treatment failures in humans, as many of these compounds showed powerful activities against antibiotic-resistant strains of bacteria and fungi such as methicillin-resistant *Staphylococcus aureus*, vancomycin-resistant bacteria, and many others. Currently, a significant portion of the novel antifungal and antibacterial drugs in clinical trials was derived from marine species, particularly bacteria as well as sponges. Given the vast number of undiscovered compounds in the oceans, all the new compounds identified might only be the tip of the iceberg, which is quite significant.

## Figures and Tables

**Table 1:** Some antifungal compounds isolated from marine bacteria.

Isolated Compound	Marine Sources	Activities	MIC µg/mL
Basiliskamides A (**7**) Basiliskamides B (**8**) [22]	*Bacillus laterosporus*	*C. albicans* *A. fumigatus*	1.0–5.0
Mohangamide A (**9**) [23]	*Streptomyces* sp.	*C. albicans*	4.14
Haliangicin (**10**) [24,25]	*Haliangium luteum*	*Botrytis cinerea* *Pythium ultimum* *Saprolegnia parasitica*	3.1 0.4 0.1
Gageostatin A (**11**) [26,27]	*B. subtilis* 109GGC020	*Rhizoctonia solani* *B. cinerea*	4
Hassallidin A (**12**) [28]	epilithic cyanobacteria	*A. fumigatus* *C. albicans*	4.8
Macrolactins T (**13**) Macrolactins B (**14**) [29]	*Bufo marinus*	*Pyricularia oryzae* *Alternaria. solani*	0.8–4.8

Compounds **7**–**14** as shown in Scheme 1

**Table 2:** Compounds with antifungal activity against *C. albicans* isolated from sea sponges.

Isolated Compound	Marine Sources	Ref.	Conc. of Inhibition
Ceratinadins A (**39**) Ceratinadins B (**40**)	*Pseudoceratina* sp.	[54,55]	MIC 2 µg/mL 4 µg/mL
Neopeltolide (**41**)	Okinawan sponge	[56,57]	MIC 0.62 µg/mL
Theonellamide G (**43**)	*Theonella swinhoei* in the red sea	[58]	IC_50_ 4.49 µM
Aurantoside K (**42**) *	*Melophlus* sp.	[59]	MIC 31.25 μg/mL
Callipeltins peptides F (**44**) Callipeltins peptides G (**45**) Callipeltins peptides H (**46**) Callipeltins peptides I (**47**) Callipeltins peptides J (**48**) Callipeltins peptides K (**49**)	*Latrunculia* sp. sponge usually found in Vanuatu islands and South Pacific	[60,61]	MIC 100 µM

* Aurantoside K has activity against wild type *C. albicans* with MIC value of 1.95 μg/mL [59]. Compounds **39**–**49** as shown in Scheme 1.

**Table 3:** Polyketide compounds which have antibacterial activity and which were isolated from marine organisms.

Isolated Compound	Marine Sources	Activities	MIC µg/mL
Ariakemicins A (**69**) [98,99]	*Rapidithrix* sp. (marine gliding bacterium)	*Brevibacterium* sp. *S. aureus* *B. subtilis*	830.4683
Ascochytatin (**70**) [100]	marine-derived fungus, *Ascochyta* sp.	*B. subtilis*	4.8
Essramycin (**71**) [101,102]	culture broth of the marine *Streptomyces* sp.	*E. coli* *P. aeruginosa* *B. subtilis* *S. aureus* *M. luteus*	1–8
Phomolides B (**72**) [103]	culture of *Phomopsis* sp.	*E. coli* strains *B. subtilis*	5–101.25
Sulfoalkylresorcinol (**73**) [104]	marine-derived fungus *Zygosporium* sp.	*S. aureus*	12.5

Compounds **69**–**73** as shown in Scheme 1.

**Table 4:** A list of some terpenes isolated from marine organisms.

Isolated Compound	Marine Sources	Terpenes Class	Activities	MIC µg/mL
Erogorgiaene (**85**) [116]	*Pseudopterogorgia elisabethae*	Serrulatane Diterpenes	*M. tuberculosis*	12.5
22-deoxyvariabilin (**86**), [117,118]	Sponge *Ircinia variabilis*	Sesterterpene	*S. aureus* *B. subtilis*	50 100
Pseudopterosin P and Q, (**87**), [119]	*Pseudopterogorgia elisabethae*	diterpene glycosides	*S. pyogenes* *S. aureus* *E. faecalis*	0.8 and 1 2 and 2.3 3.5 and 3.6
Isojaspic acid (**88**), [120]	sponge *Cacospongia*	meroditerpene	*S. epidermis*	2.5
(–)-Microcionin, (**89**) [121]	*Fasciospongia* sp.	furanosesquiterpenes	*M. luteus*	6
Sargaquinoic acid, (**90**), [122]	*Sargassum sagamianum*	Plastoquinones	*S. aureus*	2

Compounds **85**–**90** as shown in Scheme 1.

## Data Availability

Not applicable.
